# Back to the Roots: The Integration of a Constructed Wetland into a Recirculating Hatchery - A Case Study

**DOI:** 10.1371/journal.pone.0123577

**Published:** 2015-04-08

**Authors:** Miloš Buřič, Josef Bláhovec, Jan Kouřil

**Affiliations:** 1 University of South Bohemia in Ceske Budejovice, Faculty of Fisheries and Protection of Waters, South Bohemian Research Center of Aquaculture and Biodiversity of Hydrocenoses, Vodňany, Czech Republic; 2 Trout farm Mlýny, Stachy, Czech Republic; Temasek Life Sciences Laboratory, SINGAPORE

## Abstract

Aquaculture is currently one of the fastest growing food-producing sectors, accounting for around 50% of the world's food fish. Limited resources, together with climatic change, have stimulated the search for solutions to support and sustain the production of fish as a nutritious food. The integration of a constructed wetland (CW) into a recirculating hatchery (RHS) was evaluated with respect to its economic feasibility and environmental impact. The outcome of eight production cycles showed the potential of CW integration for expanded production without increased operation costs or environmental load. Concretely, the use of constructed wetland allows the rearing about 40% more fish biomass, resulting in higher production and profitability. The low requirements for space, fresh water, and energy enable the establishment of such systems almost anywhere. Constructed wetlands could enhance the productivity of existing small scale facilities, as well as larger systems, to address economic and environmental issues in aquaculture. Such systems have potential to be sustainable in the context of possible future climate change and resource limitations.

## Introduction

The projected increase in global population to 8.3 billion by 2030 [1] will be accompanied by substantial growth in food, water, and energy demands. These factors are interlinked, and issues of supply and demand must be addressed within a context of future climate change [1,2]. The increased pressure on critical food and water resources are already observable. Aquaculture is an important source of high value food [3], [4], [5] and is the fastest growing animal-based food producing sector, outpacing population growth [2,6].

Aquaculture applies increasingly sophisticated approaches to obtain high production with low water usage and wastewater production [7,8,9,10]. The core of this practice is water re-use with complex systems for treatment of recirculating water and wastewater [11,12,13,14]. The development and expansion of new technologies usually go hand in hand with increasing initial investment and high energy demands during operation [7,10]. A scheme is needed to balance economic feasibility and environmental sustainability. Currently, the emphasis is on simplification and streamlining of existing types of recirculating aquaculture systems (RAS) to expand production per surface unit while reducing initial investment, energy and water demands, and waste production and increasing waste utilization [11,13,15]. One potential solution may be the use of integrated systems [16,17,18] including constructed wetlands [19,20,21] or aquaponics [22,23] incorporated into the RAS or for effluent water treatment [24,25,26], or a combination of these approaches. Such an approach can maintain long-term environmental sustainability of aquaculture systems and production of a valuable food product [3,4].

Salmonids are one of the most important cultured fishes[2], with high nutritional and culinary value [27,28]. Among freshwater salmonids, the dominant culture species is rainbow trout (*Oncorhynchus mykiss*, Walbaum 1752) [2]. Traditional culture is usually linked to clean headwaters or, in hatcheries, to a spring fed water supply [29,30]. With the progressive demand for trout leading to increased production, the environmental impact of traditional trout culture using flow-through systems is observable: Effluent waters are high in nutrients and organic matter [26,30,31], there is increased risk of disease transfer [32,33], and escaped fish pose a threat to indigenous fauna [34,35]. In addition, the potential locations for new flow-through facilities are limited, and certain governments have imposed restrictions on production levels of existing or future farms based on the volume and content of effluent water [36,37]. This has spurred development of new technologies for RAS that reduce fresh water usage, limit disease transfer, and decrease the possibility of environmental degradation [11,38,39]. Recirculating aquaculture systems, however, involve high initial investment and energy consumption, which determines economic viability. The move to recirculating systems has thus far not reached hatcheries to a significant extent. This is unfortunate, since in the areas where springs occur and where hatcheries are generally located can be vulnerable even to low volumes of effluent water and nutrient concentrations due to reduced flow and the occurrence of vulnerable organisms specific to oligotrophic waters [15,30].

Traditional recirculating hatcheries generally involve specialized technologies for water treatment, such as microsieve filtration, ozonization, and UV sterilization [9,10,40], raising energy consumption and requiring higher initial investment and operation costs. In contrast, a less sophisticated approach involving simple construction and low operation and investment costs can allow even low capacity hatcheries to be profitable [15]. Production capacity of such systems can be increased without additional cost by integration of a small constructed wetland into the recirculating hatchery which is an economical and environmentally sound practice In consideration of the need for efficient space utilization, the primary focus of the present study was to establish the potential of a constructed wetland to increase fish production and expand profitability without additional energy demands, while decreasing the contamination of the environment by effluent water.

## Material and Methods

### Study Site

**Fig 1:**
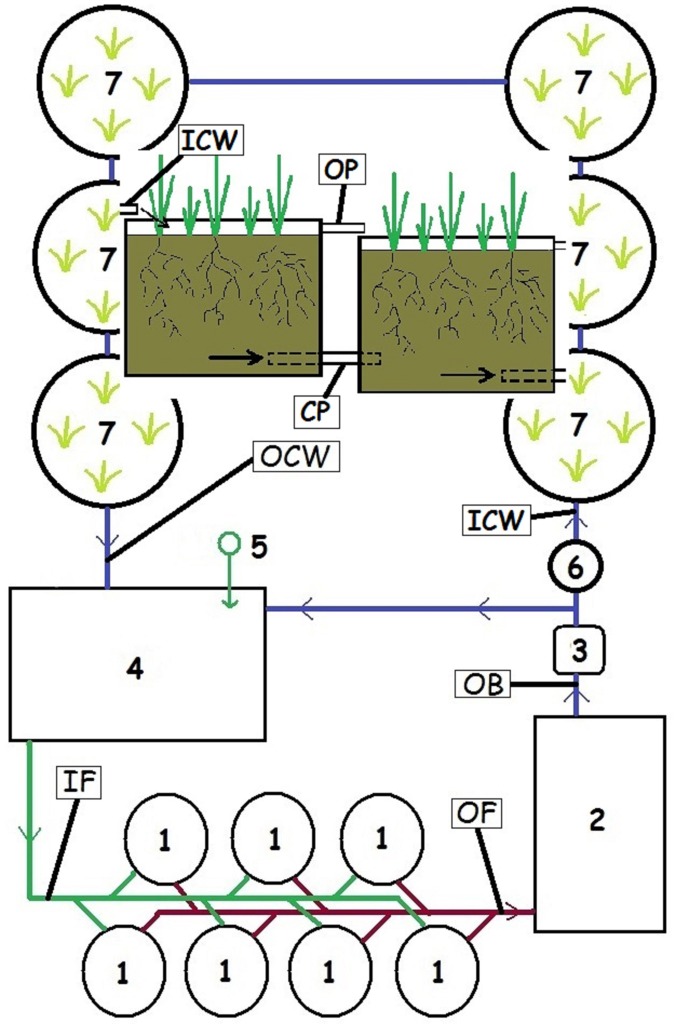
Schematic of recirculating hatchery system with the integrated constructed wetland: 1—fish tanks, 2—biofiltration/sedimentation unit, 3—circulation pump, 4—retention tank, 5—fresh water inlet, 6—ball valve, 7—constructed wetland (with the detail of water flow through tanks). Four water sampling sites are labelled: IF—inlet to fish tanks, OF—outlet from fish tanks, OB—outlet from biofilter, ICW—inlet to constructed wetland, OCW—outlet from constructed wetland, OP—outflow pipes, CP—connecting pipes for subsurface flow.

The study was conducted at a small trout farm in the Czech Republic (49°6'35"N, 13°45'10"E) where the simple recirculating hatchery system (RHS) with total energy consumption of 1.6 kW and overall fresh water demand of 0.05 L sec^-1^ was developed and tested in the past[15]. The RHS consisted of two separate systems in an area of ~65 m^2^. The first system was used for egg incubation, hatching, and rearing through the change to exogenous feeding to a fish weight of ~0.50 g. This system was the source of fry that were used for evaluation of the second system with or without an incorporated constructed wetland (CW). This second system was equipped with seven circular tanks (~0.7 m^3^); one biofiltration/sedimentation unit (~2.2 m^3^) with 12 bioblocs (EXPO-NET A/S, Denmark); one retention tank (~3.5 m^3^); and a circulation pump (0.75 kW, Wilo SE, Germany). Six tanks containing 1.4 m^3^ of inert substrate (LIAFLOR, LIAS Vintirov, Czech Republic) planted with *Phalaris arundinacea* served as the body of the CW. The tanks were arranged horizontally with a cascading flow (Fig 1). A ball valve at the height of the inlet to the CW enabled operation of the system with or without the CW, so there was no need for additional pumps or supplemental power. During operation with the CW, one third (~ 6 L s^-1^) of the total water flow was directed through the CW; hence the volume of water passing through the CW was 21.6 m^3^ h^-1^, and the total volume of the system (10.6 m^3^) passed through the CW more than twice each hour. Source of fresh water was a borehole.

The study was carried out on private land with a permission of the owner, who is included among to the article authors. No specific permissions were required for the locations and activities involved in this study. The study did not involve endangered or protected species. All experimental manipulations (rearing, capture and measurements) were conducted according to the principles of the Ethical Committee for the Protection of Animals in Research of the University of South Bohemia, Faculty of Fisheries and Protection of Waters, Research Institute of Fish Culture and Hydrobiology, Vodňany, based on the EU harmonized animal welfare act of Czech Republic. The above named Institutional Animal Care and Use Committee (IACUC) specifically approved this study. The principles of laboratory animal care and the national laws 246/1992 and regulations on animal welfare were followed (Ref. number 22761/2009-17210).

### Monitoring Physical and Chemical Conditions

Water samples from the RHS were collected bi-weekly throughout eight production cycles, four with CW and four without CW. Four sampling sites were monitored when CW was used: the inlet to fish tanks (IF), outlet from fish tanks (OF), outlet from the biofiter (OB), and outlet from CW (OCW). Samples were taken from the IF, OF, and OB when the CW was disconnected (Fig 1). All samples were analyzed in an accredited laboratory (Bioanalytika CZ, s.r.o., testing laboratory no. 1012) for ammonia (NH_4_^+^), nitrite (NO_2_-) and nitrate (NO_3_-) concentrations, biological oxygen demand (BOD), chemical oxygen demand (COD_Mn_), alkalinity (acid-neutralizing capacity), chloride concentration (Cl-), and suspended solids. Oxygen saturation level (oximeter Oxi 3205 with CellOx 325, WTW Gmbh, Weilheim, Germany), pH (pH meter pH 330i with SenTix 41, WTW Gmbh, Weilheim, Germany), and water temperature (KM12 digital thermometer, Comark Instruments, Great Britain) were monitored daily.

### Fish and Production Cycles

The study used all-female rainbow trout delivered as eyed eggs (100 000 eggs per production cycle) from certified disease-free farms (Troutex ApS, Denmark). Eggs were raised in incubation units of the first system, and approximately four days post-hatching were moved to trays for rearing to the size required for the study (0.4–0.5 g). The stocked fry were fed 5–6 times per day on pelleted feed at 3.0–5.5% of fish biomass, according to water temperature, fish size, and appetite. During the production cycles, fish weight increments were measured bi-weekly. The amount of supplied feed and mortality were recorded daily. The feed conversion ratio (FCR) was calculated to assess the utilization of feed using following formula:

$${\text{FCR = w}}_{\text{k}}{\text{/w}}_{\text{p}}$$

where w_k_ = amount of feed (kg) and w_p_ = obtained weight increment (kg).

The servicing was comparable for systems with and without CW and consisted of daily removal of faeces and dead fish and removal of sludge from biofiltration/sedimentation tank and cleaning of Bioblocs every second day. To prevent potentially high nitrite levels, sodium chloride was added to maintain Cl^-^ content at ~100 mg L^-1^. Trials were complete when mean fish weight reached 2 g. Eight production cycles were completed and monitored, four with CW realized throughout 2013 (rainbow trout eggs stocked at the end of December 2012, and in May, July, and September 2013) and four without the CW realized throughout 2012 (rainbow trout eggs stocked in January, April, June, and September 2012).

### Data Analysis

Data were analysed using Statistica 9.0 (StatSoft, Inc.). Results were first examined for normal distribution (Kolmogorov–Smirnov test) and homoscedasticity (Levene’s test). One-way ANOVA with Tukey’s post hoc test was used to analyse the data from water analyses, the Mann–Whitney test was used for basic physical and chemical parameters, and the t-test for comparisons of fish growth and feed conversion ratio. The null hypothesis was rejected at α = 0.05. Data are presented as mean ± standard deviation.

## Results

### Physical and Chemical Parameters

Oxygen saturation fluctuated during the study in the range 87–100% and 79–97% at the inlet and outlet from fish tanks, respectively. There were no significant differences among trials. Water temperature ranged from 9.2 to 12.4°C throughout the study with no significant differences among trials and showed a maximum 24 h divergence of 0.3°C. The pH values in both trials remained near optimal values for fish rearing and biofilter function. Suspended solid concentration was higher when the CW was used, but with no observed negative impact on fish (Table 1).

**Table 1:** Biomass and physical and chemical parameters observed at the outlet from fish tanks.

Parameter	O	N	Mean	STD	Min	Max
Biomass (kg)	CW	21	131.7^a^	66.6	53.7	239.9
	X	22	102.6^a^	60.7	24.7	199.7
Water temperature (°C)	CW	310	10.2^a^	1.0	9.2	12.1
	X	316	11.0^a^	1.0	9.9	12.4
pH	CW	21	7.3^a^	0.3	6.8	7,8
	X	22	7.4^a^	0.2	7.1	7.7
Suspended solids (mg L^-1^)	CW	21	6.3^a^	3.3	2.0	13.0
	X	22	3.3^b^	1.5	2.0	5.0
Chlorides (mg L^-1^)	CW	21	48.5^a^	22.7	23.6	77.2
	X	22	50.0^a^	33.4	13.7	131.0
Phosphorus (mg L^-1^)	CW	21	0.6^a^	0.3	0.2	1.1
	X	22	0.6^a^	0.6	0.1	1.5
Alkalinity (mmol L^-1^)	CW	21	1.6^b^	0.3	1.1	1.9
	X	22	1.9^a^	0.2	1.5	2.1

Data are presented as mean, standard deviation (STD), and minimum and maximum value obtained for n samples during hatchery operation (O) with constructed wetland (CW) and without constructed wetland (X).

Different alphabetic superscripts for the same parameter indicate significant differences at α = 0.05 (t-test; Mann–Whitney test).

The biofilter was adequate to deal with nitrous compounds both with and without CW integration. Significantly lower values were detected in mean ammonia concentration with CW use (Kruskal–Wallis test, H = 22.01, *p* = 0.0012), whereas the differences in mean nitrate (Kruskal–Wallis test, H = 4.28, *p* = 0.6393) and nitrite (ANOVA, H = 0.12, *p* = 0.9937) were not significant, with levels varying over a wide range (Table 2). Nevertheless, the maximum values of nitrous compounds in the terminal phase of rearing, when total biomass had reached ~230 kg and ~200 kg with and without CW integration, respectively, showed large differences between trials, with potentially lethal concentrations of ammonia and nitrites observed when the CW was not used (Tables 2 and 3). Significantly lower BOD was observed with CW (Kruskal–Wallis test, H = 15.55, *p* = 0.0164), while the differences in COD were not significant (Kruskal–Wallis test, H = 6.25, *p* = 0.3958) (Table 2). The lower values of ammonia and nitrates, as well as of BOD and COD, obtained with the CW implied a potential final maximum biomass of approximately 40% over that obtained in the system without the CW (Table 3). There were no significant differences among system sampling sites in any measured value.

**Table 2:** Chemical values at different sampling sites (inlet to fish tanks—IF, outlet from fish tanks—OF, outlet from biofilter—OB, and outlet from CW—OCW).

Parameter	O	OM	n	Mean	STD	Min	Max
Total ammonia (mg L^-1^)	CW	IF	21	0.5^a^	0.2	0.2	0.7
		OF	21	0.6^a^	0.2	0.3	0.9
		OB	21	0.5^a^	0.2	0.2	0.8
		OCW	21	0.3^a^	0.3	0.1	0.8
	X	IF	22	1.0^b^	0.7	0.1	2.2
		OF	22	1.1^b^	0.7	0.2	2.3
		OB	22	1.0^b^	0.7	0.1	2.3
Nitrite (mg L^-1^)	CW	IF	21	0.3^a^	0.2	0.1	0.8
		OF	21	0.3^a^	0.3	0.1	1.1
		OB	21	0.4^a^	0.2	0.1	0.9
		OCW	21	0.3^a^	0.3	0.1	1.0
	X	IF	22	1.0^a^	1.5	0.1	4.9
		OF	22	0.9^a^	1.3	0.1	4.3
		OB	22	1.0^a^	1.3	0.1	4.5
Nitrate (mg L^-1^)	CW	IF	21	25.2^a^	12.6	7.3	41.9
		OF	21	24.6^a^	14.3	7.9	49.8
		OB	21	25.5^a^	14.1	7.6	51.7
		OCW	21	26.0^a^	13.9	6.9	53.1
	X	IF	22	21.5^a^	24.5	5.0	73.8
		OF	22	21.1^a^	24.3	5.0	72.9
		OB	22	21.6^a^	24.8	5.0	74.0
Biological oxygen demand (mg L^-1^)	CW	IF	21	*1.0^a^	0.0	*1.0	*1.0
		OF	21	*1.0^a^	0.0	*1.0	*1.0
		OB	21	*1.0^a^	0.0	*1.0	*1.0
		OCW	21	*1.0^a^	0.0	*1.0	*1.0
	X	IF	22	1.3^b^	0.5	*1.0	2.0
		OF	22	1.4^b^	0.6	*1.0	2.5
		OB	22	1.3^b^	0.5	*1.0	2.0
Chemical oxygen demand (mg L^-1^)	CW	IF	21	1.9^a^	0.4	1.4	2.5
		OF	21	2.1^a^	0.3	1.5	2.6
		OB	21	2.0^a^	0.3	1.5	2.5
		OCW	21	2.0^a^	0.4	1.1	2.4
	X	IF	22	2.5^a^	1.0	1.1	4.0
		OF	22	2.6^a^	1.1	1.0	4.1
		OB	22	2.6^a^	1.0	1.1	4.0

Data are presented as mean, standard deviation (STD), and minimum (Min) and maximum (Max) values of the number of samplings (n) during hatchery operation (O) with constructed wetland (CW) and without constructed wetland (X).

Different alphabetic superscripts for the same parameter indicate significant differences at α = 0.05 (ANOVA, Kruskal–Wallis test).

* The values of biological oxygen demand were below laboratory detection limits (1 mg L^-1^).

**Table 3:** Ammonia and nitrite concentration and fresh water demand during operation of the recirculating hatchery system with and without constructed wetland (CW) for mean and maximum biomass, and an estimated potential maximum RHS capacity.

Operational system	Without CW		With CW	
Parameter	Mean biomass	Maximum biomass	Mean biomass	Maximum biomass
Biomass (kg)	102.6	199.7	131.7	239.4
Ammonia (mg L^-1^)	1.0	2.2	0.5	0.7
Nitrite (mg L^-1^)	1.0	4.9	0.3	0.8
Water demand (L s^-1^)	0.05		0.06	
Potential RHS capacity (kg)	~ 200		~ 280–290	

### Production Cycle and Feed Utilization

The length of the production cycle, mortality, and FCR did not differ significantly among trials (Table 4). The mean biomass was slightly higher in trials with CW, but the difference was not significant (t-test, t = 1.02, *p* = 0.3220, Table 1).

**Table 4:** Duration of Phase 1 (number of days from the beginning of egg incubation to weight ~0.50 g and Phase 2 (number of days to the weight of 2 g) and the production cycle length (number of days from the beginning of egg incubation to weight of 2 g); losses in Phase 1 (percent of dead and deformed specimens); losses in Phase 2 (the percent of dead specimens); total losses; and feed conversion ratio (FCR).

Parameter	O	n	Mean	STD
Phase 1 length (days)	CW	4	44.8^a^	2.4
	X	4	44.1^a^	3.0
Phase 2 length (days)	CW	4	32.8^a^	2.9
	X	4	35.0^a^	3.0
Production cycle length (days)	CW	4	77.5^a^	3.6
	X	4	79.0^a^	2.1
Losses in phase 1 (%)	CW	4	17.4^a^	2.7
	X	4	18.0^a^	1.3
Losses in phase 2 (%)	CW	4	2.4^a^	0.7
	X	4	3.0^a^	0.6
Total losses (%)	CW	4	19.9^a^	2.3
	X	4	21.1^a^	2.1
FCR	CW	4	0.63^a^	0.08
	X	4	0.61^a^	0.09

Data are presented as mean and standard deviation (STD) of values observed at four samplings (n) during hatchery operation (O) with (CW) and without (X) a constructed wetland.

Different alphabetic superscripts for the same parameter indicate significant differences at α = 0.05 (t-test).

### Overall Annual Production

The production cycle length and construction of RHS enables the completion of at least four production cycles per year [15]. The calculation of potential annual production of the RHS with and without CW for two levels of initial stocked biomass is presented in Table 5. Potential final biomass with CW use was estimated to be 40% greater than without CW use.

**Table 5:** Economic value of constructed wetland (CW) use in a recirculating hatchery system compared to operation without CW with different numbers of eyed eggs as initial stock per production cycle (PC).

Operation	Without CW		With CW	
Initial stock (pcs)	90000	110000	90000	110000
Total losses (%)	25	25	25	25
Number of fingerlings per PC	67500	82500	67500	82500
Final biomass (kg)	max 200	max 200	max 280	max 280
Mean weight (g)	2.96	2.42	4.15	3.39
Price of fingerlings per PC ($)*	13809	15858	15508	17595
Price of fingerlings per year ($)*	55236	63432	62032	70380

The mean final weight of fingerlings is based on the calculated possible maximum final biomass in the hatchery system. The price of fingerling is based on real prices at the study site. The annual price of fingerlings assumes four production cycles (PC) per year.

* Converted from real fingerling prices of trout farm at the study site.

### Fresh Water Demand, Energy Consumption, and Labour

The fresh water necessary to replenish that lost through evaporation and tank cleaning, with and without CW was 2.33 and 2.01 m^3^ day^-1^, respectively. The energy demand was similar in both cases, reaching an average of ~23 kWh day^-1^. Labour was also comparable, with an additional ~4 h per year required for cutting of plants in the CW at the end of the growing season and composting the biomass (Table 6).

**Table 6:** Energy consumption (kWh), freshwater demand (m^3^), and labour (hr) per day during Phase 1 and Phase 2 of the production cycle and for the entire production cycle (PC) in the recirculating hatchery system with (CW) and without (X) use of integrated constructed wetland.

	days per PC	Energy consumption		Freshwater demand		Labour	
CW		per day (kWh)	total per PC (kWh)	per day (m^3^)	total per PC (m^3^)	per day (hr)	total per PC (hr)
Phase 1	44.8	20.9	936.3	0.86	38.53	<4	<179.2
Phase 2	32.8	25.7	843.0	4.32	141.70	<3	<98.4
PC	77.5	23.0	1779.3	2.33	180.22	<3.58	<278.0
	days per PC	Energy consumption		Freshwater demand		Labour	
X		per day (kW)	total per PC (kW)	per day (m^3^)	total per PC (m^3^)	per day (hr)	total per PC (hr)
Phase 1	44.1	20.9	921.7	0.86	37.93	<4	<176.4
Phase 2	35	25.7	899.5	3.46	121.10	<3	<105.0
PC	80	23.1	1821.2	2.01	159.03	<3.56	<281.4

## Discussion

The present study addressed the efficacy of a simple technology in aquaculture. Could the evaluated system potentially increase production and decrease pollution while maintaining low energy demands and provide an effective simplified approach counter to the current technological boom? The development of technologically advanced systems overcomes former limitations on production, but is incompatible with minimizing demands on resources [1].

The use of a simple recirculating hatchery previously confirmed as effective [15] provided the opportunity to evaluate an added integrated CW under real operating conditions as a simple solution for environmentally sound culture intensification [16,41,42]. Water quality monitoring confirmed the effectiveness of the RHS, both with and without an integrated CW. The issue of primary importance for the operation of recirculating systems is the cycle of nitrous compounds [43,44,45], alterations of which can indicate malfunction of the RAS and risk to fish growth, health, and survival [46,47]. In general, biofilter function was adequate in both systems. Concentrations of ammonia and nitrites sufficiently high to affect fish stock [47] were detected only in the final phase of the production cycle when the total biomass in the RHS reached 170–200 kg in the system without CW. While the mean values of water parameters, except for BOD, did not differ, maximum concentrations with CW did not reach potentially dangerous levels as observed during operation without CW. In addition, with CW, less variation was detected for all monitored parameters except nitrates, indicating the use of CW as a tool for stabilizing conditions. This supports the feasibility of increasing biomass in systems with the CW. On the other hand, the toxic influence of high ammonia and nitrite levels was reduced by pH monitoring and adjustment, along with chloride application, enabling fish to withstand greater concentrations than those usually considered lethal [48,49].

Maintaining water quality gave consistently good production parameters, such as high growth rate and low FCR, resulting in a short production cycle. There were no observed positive or negative effects of CW use on fish growth and fitness. The shorter production cycle allowed at least four production cycles per year in both systems. The use of the CW offered the potential to increase biomass at the end of each production cycle either by rearing higher numbers of fish or by producing larger fingerlings. The latter is the preferred, because of the lower incidence of aggressive behaviour and cannibalism and better growth at lower densities [50,51,52]. Nevertheless, either approach could have important economic benefits, albeit at the possible expense of animal welfare (Table 5).

In most cases, increased production leads to higher environmental costs from effluents as well as higher water demands and energy consumption [1,30,53]. These issues, with a growing world population, form a nexus among food, water, and energy compounded by climate change [1]. Hence, new technologies and modifications for inland aquaculture are being developed to reduce its effect on freshwater ecosystems [29,39]. Large scale RAS systems have been developed that minimize fresh water demand and waste production, but with higher energy consumption [7,29]. Traditional flow-through farms, originally characterized by high fresh water demand and uncontrolled effluent discharge, currently may use lagoons [36], constructed wetlands, or aquaponic systems [22,26,54] to treat effluent water and decrease the amount of nutrients discharged into streams. Constructed wetlands and aquaponics are increasingly integrated into intensive RAS [16,42,55], but no such adaptations have been made in salmonid hatcheries, which are usually situated in spring areas with the potential to affect local oligotrophic ecosystems [30,56].

The evaluated small-scale hatchery has the potential to show a new direction of RAS simplification with integration of an environmentally sound approach for production increase. The use of a constructed wetland can provide a solution for larger systems, and can be modified to produce food plants in the CW [22,23]. It is possible that simplification of existing RAS and CW incorporation can conserve a considerable amount of energy. When considering the proportion of energy costs on the total costs, they covered only about 7% of total cost per production cycle, which can be classed as advantageous. This issue is naturally more accented in large systems for ongrowing phase. In addition, similar systems can be established almost anywhere. The on-site production of fingerlings in simple systems can have positive effects on aquaculture facility economics (cheaper fingerlings, no shipping costs), zoohygiene (fingerlings from a known source without contact with surface waters), and the environment (no transportation of fingerlings, low energy consumption, low fresh water demands, low volume of effluent water, sludge management).

Waste from the RHS with CW integration can be better managed than in flow-through aquaculture systems. In traditional flow-through hatcheries the volume of waste water is substantially higher, and treatment options are limited. The volume of effluent from RHS was lower but more concentrated [15]. This allows sludge sedimentation and collection, the potential use of sludge as fertilizer or for composting [12,57], and treatment of the remaining wastewater through additional constructed wetlands [24,25] or aquaponics [22,31] to prevent the trophic effects of effluent on ecosystems [12,56,58]. The only added waste of CW use is the plant biomass, which can be composted [59] separately or along with collected sludge [57].

## Conclusions

Constructed wetland integration has the potential to increase production of a recirculating hatchery without added operational or environmental costs. The use of a simple system with CW integration can be recommended for managing energy consumption and labour and increasing production of salmonid, or other fish species, fingerlings to supply RAS facilities and to support the development of this fast growing agriculture sector. Such systems can be sustainable, taking into account possible future climate change and increased pressure on resources.
